# Single nucleotide polymorphisms (SNPs) involved in insulin resistance, weight regulation, lipid metabolism and inflammation in relation to metabolic syndrome: an epidemiological study

**DOI:** 10.1186/1475-2840-11-133

**Published:** 2012-10-29

**Authors:** Cécile M Povel, Jolanda MA Boer, N Charlotte Onland-Moret, Martijn ET Dollé, Edith JM Feskens, Yvonne T van der Schouw

**Affiliations:** 1 National Institute for Public Health and the Environment (RIVM), Bilthoven, The Netherlands; 2 Division of Human Nutrition, Wageningen University, Wageningen, The Netherlands; 3 Julius Center for Health Sciences and Primary Care, University Medical Center Utrecht, Utrecht, The Netherlands

**Keywords:** Metabolic syndrome, Genetics, *MC4R*, *IRS1*

## Abstract

**Background:**

Mechanisms involved in metabolic syndrome (MetS) development include insulin resistance, weight regulation, inflammation and lipid metabolism. Aim of this study is to investigate the association of single nucleotide polymorphisms (SNPs) involved in these mechanisms with MetS.

**Methods:**

In a random sample of the EPIC-NL study (*n* = 1886), 38 SNPs associated with waist circumference, insulin resistance, triglycerides, HDL cholesterol and inflammation in genome wide association studies (GWAS) were selected from the 50K IBC array and one additional SNP was measured with KASPar chemistry. The five groups of SNPs, each belonging to one of the metabolic endpoints mentioned above, were associated with MetS and MetS-score using Goeman’s global test. For groups of SNPs significantly associated with the presence of MetS or MetS-score, further analyses were conducted.

**Results:**

The group of waist circumference SNPs was associated with waist circumference (P=0.03) and presence of MetS (P=0.03). Furthermore, the group of SNPs related to insulin resistance was associated with MetS score (P<0.01), HDL cholesterol (P<0.01), triglycerides (P<0.01) and HbA1C (P=0.04). Subsequent analyses showed that *MC4R* rs17782312, involved in weight regulation, and *IRS1* rs2943634, related to insulin resistance were associated with MetS (OR 1.16, 95%CI 1.02-1.32 and OR 0.88, 95% CI 0.79; 0.97, respectively). The groups of inflammation and lipid SNPs were neither associated with presence of MetS nor with MetS score.

**Conclusions:**

In this study we found support for the hypothesis that weight regulation and insulin metabolism are involved in MetS development**.***MC4R* rs17782312 and *IRS1* rs2943634 may explain part of the genetic variation in MetS.

## Background

The metabolic syndrome (MetS) is a common multi-component condition consisting of abdominal obesity, dyslipidaemia, hypertension and hyperglycaemia. It is associated with an increased risk of CVD (cardiovascular diseases) and T2D(type 2 diabetes) [1]. A central question in understanding MetS is why its underlying traits cluster together.

Several mechanisms, including insulin resistance [1], abdominal obesity [1], and inflammation [2,3] have been proposed to underlie the clustering of MetS features. However, the etiology of MetS has not been unravelled completely yet. Genetic association studies may help to better understand MetS etiology.

A systematic review of genetic association studies on MetS showed that until now most SNPs for which an association with MetS has been found were involved in lipid metabolism [4]. In a recent genome-wide association study (GWAS) even all single nucleotide polymorphisms (SNPs) associated with MetS were involved in lipid metabolism [5]. If effect sizes of SNPs involved in weight regulation and insulin resistance, pathways for which strong pathophysiological evidence exists [1], are small, these SNPs would not have been detected in GWAS, which have a low power due to adjustment for the large number of associations tested.

The SNPs for which an association with MetS has been established explain only a small part of the genetic variation in MetS [4-6]. Therefore, many more genetic variants remain to be discovered. SNPs associated with insulin resistance, abdominal obesity, inflammation and lipid levels in GWAS are likely candidates for an association with MetS itself. For several of these SNPs, however, an association with MetS has not been investigated with a candidate gene approach.

A common feature of genetic association studies is that the power to detect associations is low, because of small effect sizes and the small alpha caused by adjustment for multiple testing. A way to account for this problem is to increase the effect size and reduce the number of tests, by studying the joint effect of a group of SNPs [7]. To the best of our knowledge such an approach has not been undertaken in relation to MetS.

Therefore the aim of this study is to get more insight in the etiology of MetS, by studying associations between MetS and groups of SNPs that were found to be related to insulin resistance, abdominal obesity, inflammation or lipid levels in GWAS. For those groups of SNPs associated with MetS, we also study the association with the individual SNPs in this group.

## Methods

### EPIC-NL: Study design

In the EPIC-NL cohort the two Dutch contributions to the European Investigation into Cancer and Nutrition (EPIC) project are combined: the Prospect-EPIC and the MORGEN-EPIC (Monitoring Project on Risk Factors for Chronic Diseases) cohorts. Both cohorts were initiated in 1993.The study design of the combined cohort is described in detail elsewhere [8]. In brief, Prospect is a prospective cohort study among 17 357 women aged 49–70 who participated in a breast cancer screening program between 1993 and 1997. The MORGEN-project consists of 22 654 men and women aged 20–59 years recruited from three Dutch towns (Amsterdam, Doetinchem, and Maastricht). From 1993 to 1997, each year a new random sample of approximately 5000 individuals were examined.

Laboratory and genetic analyses were performed in a 6.5% random sample of the EPIC-NL study, in all incident T2D cases and in all incident CVD cases. In our study we only used the random sample. After exclusion of participants with missing blood samples (*n* = 157), missing values for haemoglobin A1c (HbA1C), waist circumference, high-density lipoprotein (HDL) cholesterol, systolic blood pressure, diastolic blood pressure, triglycerides or C-reactive protein (CRP) (*n* = 128), or with missing SNP data (*n*=433) the study population consisted of 1886 participants. All participants signed informed consent before study inclusion. Both studies complied with the Declaration of Helsinki. The Prospect-EPIC study was approved by the Institutional Review Board of the University Medical Center Utrecht and the MORGEN project was approved by the Medical Ethical Committee of TNO, The Netherlands.

### Baseline measurements

At baseline, a physical examination was performed and non-fasting blood samples were drawn. During the physical examination, systolic and diastolic blood pressure measurements were performed twice in the supine position on the right arm using a BosoOscillomat (Bosch & Son, Jungingen, Germany) (Prospect) or on the left arm using a random zero sphygmomanometer (MORGEN). The mean of both measurements was taken. Waist circumference and height were measured to the nearest 0.5 cm. Body weight was measured with light indoor clothing without shoes on, to the nearest 100 gr.

#### Biomarker measurements

HbA1c was measured with a homogeneous assay with enzymatic endpoint in erythrocytes. Trigycerides were measured in EDTA plasma using enzymatic methods, whereas hsCRP was measured with a turbidimetric method [8]. MetS was defined according to an adapted version of the AHA/NHLBI MetS definition as having at least 3 of the following 5 MetS features [9]: abdominal obesity (waist circumference ♂≥102 cm; ♀≥ 88 cm); low HDL cholesterol (♂ <1.0; ♀<1.3 mmol/L); hypertriglyceridemia ( ≥1.7 mmol/L); hypertension ( ≥130/85 mm Hg or use of hypertensive medicication); hyperglycemia (HbA1C ≥5.7% or glucose lowering medication) [10,11]. MetS-score was calculated by summing the number of MetS features present in each participant.

#### Genotyping

Genomic DNA was extracted in different batches using standard methods, such as salting out, QIAamp® Blood Kit (Qiagen Inc., Valencia, CA, USA). The participants were genotyped using a gene-centric 50K iSelect chip array, further referred to as IBC array [12]. The design and coverage of the IBC array compared to conventional genome-wide genotyping arrays has been described in detail elsewhere [12]. Additionally, the *MC4R* rs17782313SNP was genotyped in 853 women of the random sample with the KASPar chemistry, an allele-specific PCR SNP genotyping that uses FRET quencher cassette oligos [13]. For this SNP genomic DNA was extracted with an in-house developed extraction method at Kbiosciences (Hoddesdon Herts, UK).

From the available SNPs, we selected those significantly associated (P≤1.0*10^-5^) with waist circumference, inflammatory markers, triglycerides or HDL cholesterol or homeostasis model assessment insulin resistance (HOMA-IR) in published GWAS until 01-01-2011. As only a few GWAS on HOMA-IR are conducted, we additionally included SNPs both associated with a glucose related traits in GWAS (P≤1.0*10^-5^) and with HOMA-IR (P≤0.05). Highly correlated proxy SNPs were included in case the original SNP from the GWAS was not available on the IBC array (r^2^_CEU 1000 Genome Pilot 1_ ≥ 0.80). If SNPs were only found in one GWAS, without a replication sample, they were excluded. In total we included 39 SNPs: 2 SNPs associated with waist circumference, 5 SNPs associated with insulin resistance, 6 SNPs associated with inflammation, 16 SNPs associated with triglycerides and 16 SNPs associated with HDL cholesterol (Table 1). Sixteen SNPs which were associated in GWAS with waist circumference, inflammatory biomarkers and lipid levels were not on the IBC CVD array (Appendix I).

**Table 1:** SNPs included in the analyses of random sample of EPIC-NL (n=1886)

**Gene**	**SNP (literature)**	**SNP (dataset)**	**MAF (dataset)**	**ref**	**r**^**2**^**SNPs**
Insulin resistance					
*PPARG*	rs1801282	rs1801282	G: 0.13	[14]	-
*IRS1*	rs2943634	rs2943634	A: 0.35	[15]	-
*GCKR*	rs780094	rs780094	T: 0.37	[14]	-
*IGF1*	rs35767	rs35767	A: 0.16	[14]	-
*GCK*	rs4607517	rs1799884	T: 0.18	[14]	1
Abdominal obesity					
*FTO*	rs1421085	rs1421085	C: 0.40	[16]	-
*MC4R*^1^	rs17782313	rs17782313	C: 0.25	[16]	-
Inflammation					
*IL6R*	rs4537545	rs4537545	T: 0.39	[17]	-
*LEPR*	rs6700896	rs1805096	A: 0.38	[17]	0.89
*CRP*	rs7553007	rs1341665	A: 0.32	[17]	1
*ADIPOQ*	rs1648707	rs182052	A: 0.34	[18]	1
*IL18*	rs1834481	rs5744256	G: 0.26	[19]	1
*GCKR*	rs780094	rs780094	T: 0.37	[20]	-
Triglycerides					
*AFF1*	rs442177	rs3775214	G: 0.43	[21]	0.96
*APOB*	rs673548	rs673548	A: 0.22	[22]	-
*APOB*	rs693	rs693	G: 0.50	[23]	-
*APOA5-A4-C3-A1*	rs12286037	rs12286037	T: 0.08	[24]	-
*APOA5*	rs6589566	rs2075290	C: 0.06	[25]	1
*FADS1*	rs174548	rs174548	G: 0.29	[21]	
*FADS1-2-3*	rs174547	rs174577	A: 0.35	[26]	1
*GALNT2*	rs4846914	rs4846914	G: 0.41	[23]	-
*LPL*	rs328	rs328	G: 0.10	[23]	-
*MLXIPL*	rs17145738	rs17145750	T: 0.16	[24]	0.86
*PLTP*	rs7679	rs6073952	A: 0.20	[26]	0.82
*TRIB1*	rs2954029	rs2954029	T: 0.47	[26]	-
*CLIP2*	rs16996148	rs16996148	T: 0.10	[24]	-
*GCKR*	rs780094	rs780094	T: 0.37	[27]	-
*ANGPTL3-DOCK7*	rs1748195	rs1748197	A: 0.35	[24]	1
*ANGPTL3-DOCK7*	rs12130333	rs12130333	T: 0.24	[23]	-
HDL cholesterol					
*ABCA1*	rs1883025	rs1883025	T: 0.24	[26]	-
*ABCA1*	rs3890182	rs3890182	A: 0.10	[21]	-
*APOB*	rs11902417	rs11902417	A: 0.24	[21]	-
*CETP*	rs1800775	rs1800775	A: 0.46	[28]	-
*CETP*	rs3764261	rs3764261	A: 0.31	[29]	-
*FADS1*	rs174548	rs174548	G: 0.29	[21]	-
*FADS1-2-3*	rs174547	rs174577	A: 0.35	[26]	-
*GALNT2*	rs4846914	rs4846914	G: 0.41	[23]	-
*LCAT*	rs255052	rs255052	A: 0.17	[24]	-
*LCAT*	rs12449157	rs1109166	C: 0.18	[21]	0.94
*LIPC*	rs1800588	rs1800588	T: 0.22	[23]	-
*LIPG*	rs2156552	rs2156552	A: 0.16	[21]	-
*LPL*	rs328	rs328	G: 0.10	[23]	-
*PLTP*	rs7679	rs6073952	A: 0.20	[26]	0.82
*MMAB MVK*	rs2338104	rs10774708	A: 0.47	[26]	1
*HNF4A*	rs1800961	rs1800961	T: 0.04	[26]	-

^1^Data available in 853 women.

### Statistics

Distributions of genotypes were tested for deviation from hardy-weinberg equilibrium (HWE) by chi-square analyses. Triglycerides and hsCRP were log-transformed to improve normality. Participants on blood pressure medication were excluded from the analyses on blood pressure, participants on glucose lowering medication from the analysis on HbA1C, and participants with acute inflammation (hsCRP > 10 mmol/L) from the analyses on hsCRP.

**Figure 1:**
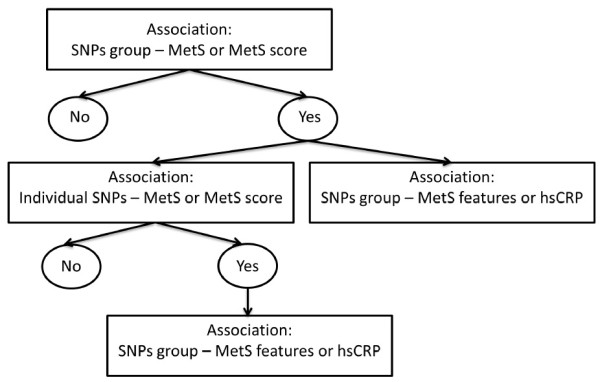
Flow diagram of analyses.

SNPs were divided into 5 groups (Table 1) according to the known associations in GWAS. These groups of SNPs were associated with the corresponding phenotype using the linear regression model of Goeman’s global test [7]. Subsequently, for each group of SNPs the association with MetS was analysed using the log-linear model of Goeman’s global test, and the association with MetS-score using the linear regression model of Goeman’s global test (see Figure 1: data-analyses scheme). If one of these associations was statistically significant, we conducted additional data-analyses. First, to see whether the association with the group of SNPs was mediated by the corresponding phenotype from GWAS, we adjusted the association between MetS and the group of SNPs for this phenotype. Second, we tested if the group of SNPs was also associated with the individual MetS features or hsCRP using the linear regression model of Goeman’s global test. Third, we analysed the association of the individual SNPs in this group with MetS using log-linear models and with MetS-score using linear regression. For the individual SNPs which were significantly associated with MetS or MetS-score, we analysed associations with the individual MetS features and hsCRP using linear regression. We only conducted analyses with individual SNPs, for those SNPs which were on the group level associated with MetS or MetS-score. Consequently, only a few associations with individual SNPs were analyses. Therefore, adjustment for multiple testing is not appropriate.

All analyses were adjusted for age, sex and cohort. Significance was defined as a 2-sided P-value <0.05. The global test was calculated in R version 2.12.1 (R Foundation for Statistical Computing; http://www.r-project.org). The analyses for individual SNPs were performed with SAS version 9.2 (SAS Institute, INC., Cary, North Carolina).

## Results

All SNPs were in HWE (P>0.05). Minor allele frequency of the SNPs ranged from 0.04-0.47 (Table 1). The random sample of EPIC-NL consisted of 465 men and 1421 women (Table 2). The mean age was 50.1 (SD 11.7) and 30.3% of all participants had MetS.

**Table 2:** Characteristics of the random sample of EPIC-NL (n=1886)

	**Total (n=1886)**	**Men (n=465)**	**Women (n=1421)**
Sex (% men)	24.6 (465)		
Age (yr)	50.1 (11.7)	43.9 (11.1)	52.2 (11.2)
Waist circumference (cm)	85.5 (11.6)	92.0 (11.4)	82.9 (10.5)
Abdominal obesity(%)^a^	27.7(522)	21.9 (102)	29.6 (420)
HbA1C (%)	5.46 (0.69)	5.27 (0.61)	5.53 (0.71)
Hyperglycemia^a^	28.3 (534)	17.8 (83)	31.7 (451)
Diabetic medication(%)	1.2 (22)	0.2 (1)	1.5 (21)
HDL-cholesterol (mmol/L)	1.27 (0.35)	1.14 (0.28)	1.31 (0.36)
Low HDL-cholesterol(%)^a^	47.8 (902)	29.2 (136)	53.9 (766)
Triglyceride (mmol/L) ^b, c^	1.32 (0.91-1.98)	1.72 (1.15-2.40)	1.22 (0.85-1.80)
Hypertriglyceridemia ^a, b^	33.8 (637)	50.8 (236)	28.2 (401)
Systolic blood pressure (mm Hg)	126.9 (18.5)	127.1 (14.8)	126.8 (19.5)
Diastolic blood pressure (mm Hg)	78.2 (10.4)	80.3 (10.1)	77.5 (10.4)
Hypertension(%)^a^	45.6 (860)	47.1(219)	45.1 (641)
Blood pressure lowering medication (%)	10.7 (202)	5.6 (26)	12.4 (176)
High sensitive CRP (mmol/L)	1.41 (0.62-3.39)	1.20 (0.53-2.82)	1.49 (0.66-3.70)
MetS-score (number of features)	1.8 (1.4)	1.7 (1.3)	1.9 (1.4)
MetS prevalence(%)^a^	30.3 (572)	25.2 (117)	32.0 (455)

Data are presented as means (standard deviation), median with inter-quartile range or % (n); *HbA1C* haemoglobin A1c, *MetS* metabolic syndrome, *hsCRP* high sensitive C-reactive protein.

^a^Abdominal obesity, low HDL cholesterol, hypertension, hypertriglyceridemia and MetS are defined according to the criteria of AHA-NHLBI (2005). Hyperglycemia is defined according to the criteria of the American Diabetes Association (2010). Abdominal obesity: ♂ ≥102 cm; ♀≥ 88 cm; Low HDL: ♂ <1.0; ♀<1.3 mmol/L; Hypertriglyceridemia: ≥1.7 mmol/L; Hypertension: ≥130/85 mm Hg or use of hypertensive medication; Hyperglycemia: HbA1C in National Glycohemoglobin Standarization Program (NGSP) units ≥ 5.7% or glucose lowering medication; MetS is defined as having at least 3 MetS features.

^b^Non-fasting values.

^c^No information on lipid lowering medication is available.

The group of abdominal obesity SNPs was significantly associated with waist circumference (P=0.01), the group of insulin resistance SNP with HbA1C (P=0.04) and the group of inflammation SNPs with hsCRP (P=7.3*10^-6^). In contrast, the group of triglyceride SNPs was not significantly associated with serum triglycerides (p=0.08) and the group of HDL cholesterol SNPs not with HDL cholesterol (P=0.32).

P-values for the association of all groups of SNPs with MetS or MetS-score are shown in Table 3. The group of SNPs known for their association with insulin resistance, was borderline significantly associated with MetS (P=0.06) and statistical significantly associated with MetS-score (P=0.003). This group of SNPs was also significantly associated with HbA1C, triglycerides and HDL cholesterol (Table 3). The associations of this group of SNPs with MetS-score and MetS features weakened slightly after adjustment for HbA1C (Table 3). Of the five insulin resistance SNPs included in the group *IRS1* rs2943634 was the only SNP individually associated with MetS or MetS-score (Table 4). These associations remained after adjustment for HbA1C (data not shown). *IRS1* rs2943634 was also associated with HbA1C (per allele difference −0.034, 95% CI −0.070; 0.002), triglycerides (per allele difference −0.051, 95% CI −0.085; -0.017) and HDL cholesterol (per allele difference 0.029, 95% CI 0.008; 0.052).

**Table 3:** P-values for Goeman’s global test, testing the statistical significance of associations of waist circumference and insulin resistance SNPs with metabolic syndrome and related features

**Group of SNPs**	**MetS**	**MetS-score**	**WC (cm)**	**HbA1C (%)**	**Log (TG) (mmol/L)**	**HDL (mmol/L)**	**SBP (mm HG)**	**DBP (mm HG)**	**Log (CRP) (mmol/L)**
*n*	*1886*	*1886*	*1886*	*1864*^*1*^	*1886*	*1886*	*1684*^*3*^	*1684*^*3*^	*1683*^*2*^
*WC*^*4*^	**P=0.03**	P=0.08	**P=0.01**	P=0.73	P=0.81	P=0.36	P=0.29	P=0.11	P=0.22
*Adj WC*	P=0.16	P=0.80	-	P=0.47	P=0.55	P=0.09	P=0.36	P=0.34	P=0.68
*IR*	P=0.06	**P=0.003**	P=0.45	**P=0.04**	**P=0.0003**	**P=0.0005**	P=0.07	P=0.16	P=0.16
*Adj HbA1C*	P=0.12	**P=0.01**	P=0.70	-	**P=0.0005**	**P=0.0008**	P=0.10	P=0.22	P=0.17

All analyses are adjusted for age, sex and cohort; *Mets* Metabolic syndrome, *WC* waist circumference, *TG* triglycerides, *HDL* HDL-cholesterol, *HbA1C* haemoglobin A1c, *SBP* systolic blood pressure, *DBP* diastolic blood pressure, *Adj*, adjusted, *IR* insulin resistance.

^1^Subjects which are using glucose lowering medication are excluded.

^2^Subjects with CRP > 10 mmol/L are excluded.

^3^Subjects with blood pressure lowering medication are excluded.

^4^Data available in 853 women.

**Table 4:** Individual SNPs associated with waist circumference or insulin resistance in GWAS in relation to MetS and MetS-score

	**MetS**	**MetS-score**
*n*	1886	1886
*WC*		
FTO rs1421085	1.02(0.93; 1.12)	0.05(−0.03; 0.14)
MC4Rrs17782313 ^1^	**1.16(1.02; 1.32)**	0.10(−0.05; 0.24)
*IR*		
PPARG rs1801282	1.04(0.91; 1.19)	0.10(−0.02; 023)
IRS1 rs2943634	**0.88(0.79; 0.97)**	**−0.14(−0.23;-0.06)**
GCKR rs780094	0.99(0.89; 1.09)	0.05(−0.04; 0.13)
IGF1 rs35767	1.03(0.91; 1.16)	0.03(−0.08; 0.14)
GCK rs1799884	1.07(0.95; 1.20)	0.08(−0.03; 0.22)

Data are presented as PR per minor allele for MetS and as minor allele change for MetS Score; *Mets* Metabolic syndrome, *WC* waist circumference, *IR* insulin resistance.

All analyses are adjusted for age, sex and cohort.

^1^Data available in 853 women.

The group of SNPs known for their association with waist circumference, was statistical significantly associated with MetS (P=0.03) and tended to be associated with MetS-score (P=0.08). The association with MetS and the suggested association with MetS-score disappeared after adjustment for waist circumference (Table 3). Furthermore, no association was found with any individual MetS feature except for waist circumference (Table 3). Of the two abdominal obesity SNPs only *MC4R* rs17782313 was individually associated with MetS (Table 4). This association remained after adjustment for waist circumference.

*MC4R* rs17782313 was not associated with any individual MetS feature, including waist circumference itself (data not shown). The groups of SNPs linked in GWAS with inflammation, triglycerides or HDL cholesterol were neither associated with MetS nor with MetS-score (all P-values ≥ 0.15). Therefore no further data-analyses were done for these groups of SNPs.

## Discussion

In this population based study of 1886 participants, we studied the relation between MetS and groups of SNPs associated in GWAS with waist circumference, insulin resistance, inflammation, triglycerides or HDL cholesterol. Only the group of waist circumference SNPs and the group of insulin resistance SNPs were associated with MetS or MetS-score.

### Waist circumference SNPs

In our study the group of SNPs which were associated with waist circumference in GWAS *(MC4R* rs17782313 and *FTO* rs1421085) was associated with waist circumference, as well as with MetS. The association with MetS disappeared after adjustment for waist circumference, indicating that the association with MetS is driven by the association with waist circumference. Furthermore, the association with MetS seemed mainly to be driven by *MC4R* rs17782313. In the KORA study among 7888 adults, an association between *MC4R* rs2229616 (r^2^=1 with rs17782313) and MetS was found [30], supporting our findings. Unfortunately, in our study, data on *MC4R* rs17782313 were available for women only. However, since in the KORA study [30] the association between *MC4R* rs2229616 and MetS was not dependent on sex, we expect that this did not influence our findings. In our study the association between *MC4R* rs17782313 and MetS remained after adjustment for waist circumference. Furthermore, we found no association between *MC4R* rs17782313 and any individual MetS feature, including waist circumference. This suggests that the association between *MC4R* rs17782313 and MetS, is at least in part, independent of body weight. In both human and animal studies *MC4R* rs17782313 had an effect on insulin resistance, independent of body weight [31,32]. Therefore, insulin resistance may explain part of the association between *MC4R* rs17782313 and MetS. Contrary to a meta-analysis among 12,555 Europeans, in which *FTO* rs9939609 (r^2^=1 with rs1421085) was significantly associated with MetS (OR 1.17 ; 95% CI 1.10-1.25) [33], we did not observe an association between *FTO* rs1421085 and MetS. This discrepancy may be explained by the weak association between *FTO* rs1421085 and waist circumference in our study. In our study the regression coefficient between *FTO* rs1421085 and waist circumference was 0.03 per SD, whereas in other studies it ranged from 0.07 per SD to 0.14 per SD [33].

### Insulin resistance SNPs

We found an association between insulin resistance SNPs and MetS and MetS-score that remained after adjustment for HbA1c, indicating that this association was not driven by HbA1C. However, since HbA1C is not an optimal marker of insulin resistance (r^2^ between HOMA-IR – HbA1C ≈0.50 [34]), we can not rule out that insulin resistance mediates this association. Out of the group of five insulin resistance SNPs, *IRS1* rs2943634 was the only SNP significantly associated with MetS and MetS score. It was also associated with HbA1C, triglycerides and HDL cholesterol. Accordingly an IRS1 knock-out mouse model displayed a MetS like phenotype with insulin resistance, increased blood pressure, increased triglycerides, decreased HDL cholesterol and decreased LPL activity [35]. Furthermore, in human studies *IRS1* rs2943634 has been associate with glucose related [15] and lipid traits [36]. In contrast, in a study among 1126 non-Hispanic whites, 898 non-Hispanic blacks and 906 Mexican Americans, *IRS1* rs7578326 (*r*^2^ with rs2943634=0.82) was not associated with MetS, neither in the overall population, nor in specific ethnic groups [37]. However, as the number of Caucasian participants and MetS prevalence were lower than in our study, the former study may have been underpowered in Caucasian. Besides *IRS1* rs2943634 the group of insulin resistance SNPs consisted of *PPARG* rs1801282, *GCKR* rs780094, *GCK*rs1799884, and *IGF1*rs35767. In line with other studies, none of these SNPs were associated with MetS in our data [4,37,38]. This may be explained by the relatively weak effect on HOMA-IR of *PPARG* rs1801282, *GCK* rs1799884, and *IGF1* rs35767 [14,15] or by pleiotropic effects of *PPARG* rs1801282 and *GCKR* rs780094. The *12Pro* allele of *PPARG* rs1801282 has opposite effects on insulin resistance and BMI in Caucasian subjects [39,40], whereas *GCKR* rs780094 has opposite effects on insulin resistance and lipid levels [27]. These opposite effects may result in a zero association with MetS, as observed by for example Passaro et al. [40].

### Lipid SNPs

We did not observe an association between groups of SNPs known for their association with triglycerides or HDL cholesterol and MetS. On the contrary, in a GWAS [5] and a systematic review of genetic association studies [4], the majority of SNPs associated with MetS was involved in lipid metabolism. The lack of an association with MetS may be a power issue, because the association between lipid SNPs and lipid levels was relatively weak in EPIC-NL. Subgroup analyses revealed that these weak associations were consistent for all lipid SNPs and could not be explained by medication use, sex or a difference between the MORGEN and Prospect study. Furthermore, it is unlikely that the non-fasting state of our samples gives an explanation, as in a GWAS, the association with lipid levels was independent of the fasting state for most SNPs [41].

### Inflammation SNPs

We found no significant association between a group of inflammation SNPs and MetS. Accordingly, in a study among 4286 British women, a *CRP* haplotype was not associated with the individual features of MetS [42]. Furthermore, Rafiq et al. could not detect an association between T2D, an endpoint of MetS and 8 SNPs known to alter circulating levels of inflammatory proteins, which were located in the *IL-18*, *IL1RN*, *IL6R*, *MIF*, *PAII* and *CRP* genes [43]. Overall, this evidence may suggest that genetic variants in inflammatory genes do not play a causal role in MetS development. However, for several reasons it cannot be ruled out that some SNPs in inflammatory pathways are causally related to MetS. First, for some inflammatory proteins SNP-MetS associations have not been investigated yet. Second, as the global test gives a combined result for all SNPs, the global test may be not significant despite the presence of an association between one of the single SNPs and MetS.

In this study we have explored the biomarkers involved in MetS development, by studying SNPs related to these biomarkers. Advantage of this approach is that according to the principles of Mendelian randomization the associations we investigated are neither affected by reverse causality nor by socioeconomic and behavioural confounders [44]. Furthermore, as all participants were Caucasian, it is unlikely that our study results have been affected by population stratification. However, for some SNPs we measured, like *IRS1* rs2943634 [45], allele frequencies are very heterogeneous among different populations. Therefore, our results warrant replication in other study populations. Sixteen SNPs which were associated in GWAS with MetS related traits were not on the IBC CVD array. Inclusion of the three waist circumference SNPs, which were not on the array, would probably have increased the possibility to find associations with several MetS features. As the global test of inflammation SNPs on hsCRP was already highly significant (P=7.3*10^-6^), inclusion of additional SNPs, which were absent on the IBC CVD array, would not have changed our results for the global test, but may have revealed additional individual SNPs. The total number of lipid SNPs in our study was relatively large and relatively few lipid SNPs were missing. Therefore we believe that inclusion of additional lipid SNPs would not have changed our results considerably on the group level. The IBC CVD array covered all insulin resistance SNPs discovered in GWAS. However, up till now for only three SNPs a genome wide association with HOMA-IR has been found and replicated. To increase power we also included those SNPs associated with glucose related traits in GWAS, which were also associated with HOMA-IR (P≤0.05). However, as the association between the group of insulin resistance SNPs and HbA1C was just significant, the power to detect associations with MetS and its features was still low.

In conclusion, we found that SNPs associated with waist circumference or insulin resistance in GWAS were also associated with MetS. These results are in line with the hypotheses that weight regulation and insulin metabolism are causative factors for MetS.

Individual SNPs for which we found an association with MetS were *MC4R* rs17782312 which is involved in weight regulation and *IRS1* rs2943634 which is involved in insulin resistance.

## Appendix I

Loci related to waist circumference, insulin resistance, inflammatory biomarkers, triglycerides and HDL cholesterol in genome wide association studies till 01-01-2011 which are not on the IBC CVD array.

## Waist circumference

NRXN3 – rs10146997 [46]

TFAP2B – rs987237 [16]

MSRA – rs7826222 [16]

## Insulin resistance

### Inflammatory biomarkers

HNF1A – *rs1183910*[20]

ARL15 – rs4311394 [18]

APOE, APOC1, APOCII – rs4420638 [47]

CDH13 – rs3865188 [48]

## Triglycerides

LPL – rs326 [49]

APOA1 – rs2075292 [49]

APOA1, APOC3, APOA4, APOA5 – rs10892151 [50]

APOA1, APOC3, APOA4, APOA5 – rs4938303[21]

CLIP2 – rs7557067 [26]

## HDL cholesterol

CETP – rs9989419 [21]

LIPC – rs10468017 [26]

CLIP2 – rs2304130 [21]

MAB,MVK – rs9943753 [21]

## Abbreviations

CVD: Cardiovascular diseases; GWAS: Genome-wide association study; HbA1C: Haemoglobin A1c; HDL: High-density lipoprotein; HWE: Hardy-weinberg equilibrium; HOMA-IR: Homeostasis model assessment insulin resistance; hsCRP: High sensitive C-reactive protein; MetS: Metabolic syndrome; SNP: Single nucleotide polymorphism; T2D: Type 2 diabetes.

## Competing interests

The authors declare that they have no competing interests.

## Authors’ contribution

CMP analyzed the data, contributed to the discussion and wrote the manuscript. JMAB contributed to the discussion and reviewed the manuscript. NCO contributed to the discussion and reviewed the manuscript. MET contributed to the discussion and reviewed the manuscript. EJMF researched the data, contributed to the discussion and reviewed the manuscript. YTS contributed to the discussion and reviewed the manuscript. All authors read and approved the final manuscript.

## Funding

The EPIC-NL study was funded by the “Europe against Cancer” Program of the European Commission (SANCO), the Dutch Ministry of Health, the Dutch Cancer Society, the Netherlands Organisation for Health Research and Development (ZonMW), and World Cancer Research Fund (WCRF).
